# Probing and controlling coherent and incoherent dynamics of phase transitions via multipulse excitation

**DOI:** 10.1126/sciadv.aef8585

**Published:** 2026-06-17

**Authors:** Feng-Wu Guo, Wen-Hao Liu, Zhi Wang, Zhong-Ming Wei, Shu-Shen Li, Lin-Wang Wang, Jun-Wei Luo

**Affiliations:** ^1^ State Key Laboratory of Semiconductor Physics and Chip Technologies, Institute of Semiconductors, Chinese Academy of Sciences, Beijing 100083, China.; ^2^ Center of Materials Science and Optoelectronics Engineering, University of Chinese Academy of Sciences, Beijing 100049, China.

## Abstract

Ultrafast laser pulses can selectively induce either wide-sized coherent or localized incoherent dynamics in solids, yet harnessing these complex dynamics to achieve controlled phase transitions remains challenging. Here, we use real-time time-dependent density functional theory (rt-TDDFT) to investigate a double-pulse laser scheme in VO_2_, elucidating the roles of these two distinct types of dynamics and proposing two more energy-efficient phase transition routes. In the first scenario, a weak initial pulse induces coherent oscillations of V-V dimers. When the second pulse is applied at the dimer stretching time, transient bandgap narrowing enhances carrier excitation, thereby reducing the total laser fluence required for the phase transition. In the second scenario, a stronger but subthreshold first pulse activates localized structural distortions resembling photoinduced polarons. A subsequent long-wavelength pulse promotes accumulation and spatial propagation of the polarons, ultimately triggering a global phase transition. Our findings establish multipulse excitation as an energy-efficient and general approach for controlling structural phase transitions and disentangling distinct ultrafast dynamics in solids.

## Teaser

Tailored pulse sequences control electronic excitation to direct phase transition pathways, enabling energy-efficient transitions.

## INTRODUCTION

Ultrafast multipulse laser techniques have long served as powerful tools for probing and manipulating coherence in matter, with early studies focusing on both electronic quantum interference (*1*–*5*) and vibrational coherence (*6*–*12*) in molecular systems. For example, the initial pulse establishes coherent molecular motion, while subsequent pulses steer the system along specific reaction pathways, thereby enhancing efficient conformational transitions (*6*–*11*). Inspired by these advances in femtochemistry, recent experiments in solids (*13*–*15*) have demonstrated that double-pulse laser excitation with a tunable delay between the two pulses provides a promising strategy to control complex dynamics and to drive phase transitions with improved energy efficiency.

However, compared to molecular systems, the long-range periodicity in solids gives rise to high electronic and phonon densities of states (*14*), which can enhance strong electron-phonon coupling and drive far more intricate dynamical phenomena. Multipulse laser processes in solids thus involve not only the control of collective coherent motions (*14*, *15*) but also the regulation of localized incoherent distortions (*13*), leading to richer behaviors. Current interpretations of multipulse dynamics are often cast in terms of simplified effective potential energy surfaces (PESs), where the first pulse lowers the barrier for a subsequent phase transition, thereby enabling the second pulse to drive the transformation more efficiently. However, the PES framework typically relies on adiabatic approximation, which assumes that faster electronic dynamics adiabatically determine to the slower ionic motion. Such treatments often neglect the complex nonequilibrium dynamics and the time-delay correlation between the multiple laser pulses, thereby limiting our ability to understand and control dynamical processes in photoinduced phase transitions (PIPTs).

**Fig. 1.**
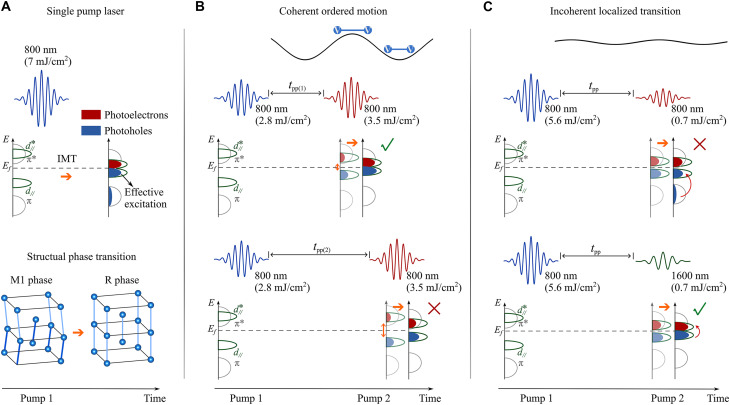
PIPTs in VO_2_ driven by single- and double-pulse excitations. (**A**) Monoclinic (M1)–to–rutile (R) structural transition associated with the insulator-to-metal transition (IMT) under a single 800-nm pump pulse (7 mJ/cm^2^). In the M1 phase, darker blue lines indicate shorter, dimerized V-V bonds. In the R phase, all V-V bonds become equivalent, and the bandgap closes. Oxygen atoms are omitted for clarity. The density of states shows the distribution of photoexcited holes (blue) and electrons (red). (**B**) Coherent structural oscillations triggered by a first 800-nm pulse (2.8 mJ/cm^2^) and followed by an M1-to-R phase transition induced by a second 800-nm pulse (3.5 mJ/cm^2^) at different delay times. (**C**) Incoherent local structural transition induced by a first 800-nm pulse (5.6 mJ/cm^2^), followed by an M1-to-R phase transition induced by a second 1600-nm pulse (0.7 mJ/cm^2^) or 800-nm pulse (0.7 mJ/cm^2^).

Here, we use real-time time-dependent density functional theory (rt-TDDFT) simulations to investigate how double-pulse excitation controls coherent and incoherent dynamics in PIPTs of VO_2_, reducing the required laser fluence by ~10% relative to single-pulse excitation. Our results reveal that tuning the fluence of the first pulse enables selective induction of either coherent collective structural oscillations (2.8 mJ/cm^2^, 800 nm) or incoherent local transitions (5.6 mJ/cm^2^, 800 nm). In the first case, the first pulse induces coherent periodic oscillations of all the V-V dimers (short bonds), with the average dimer elongation narrowing the bandgap and contraction widening it. The efficiency of carrier excitation is strongly enhanced when the second pulse (3.5 mJ/cm^2^, 800 nm) arrives during a bandgap minimum time, leading to an increased density of photoexcited holes and a more efficient phase transition compared to the single pulse case. In contrast, if the second pulse is applied when the V-V dimers reach maximum contraction (i.e., at the bandgap maximum time), then the excitation is less effective, resulting in reduced transition efficiency (Fig. 1B). In the second case, no pronounced collective oscillations in either atomic or electronic structure are observed, and the bandgap nearly collapses due to localized structural distortions, resembling polarons. Under this condition, a second 800-nm pulse (0.7 mJ/cm^2^) predominantly excites electrons from deeper valence states that are less involved in driving the transition (Fig. 1C), having an opposite effect of causing the phase transition, hence reducing the overall transition efficiency. In contrast, if a longer-wavelength 1600-nm second pulse is applied, it can more selectively excite transition-relevant electronic states, enhancing the formation and propagation of localized polarons, causing them to coalesce into a global phase transformation. These findings provide a microscopic framework linking structural dynamics and electronic excitation pathways to phase transition efficiency, offering design principles for energy-efficient control of PIPTs in phase transition materials. Furthermore, we demonstrate how multipulse laser experiments can be used to probe and disentangle different ultrafast dynamics.

## RESULTS

### Photoinduced dynamics under single laser pulse

**Fig. 2.**
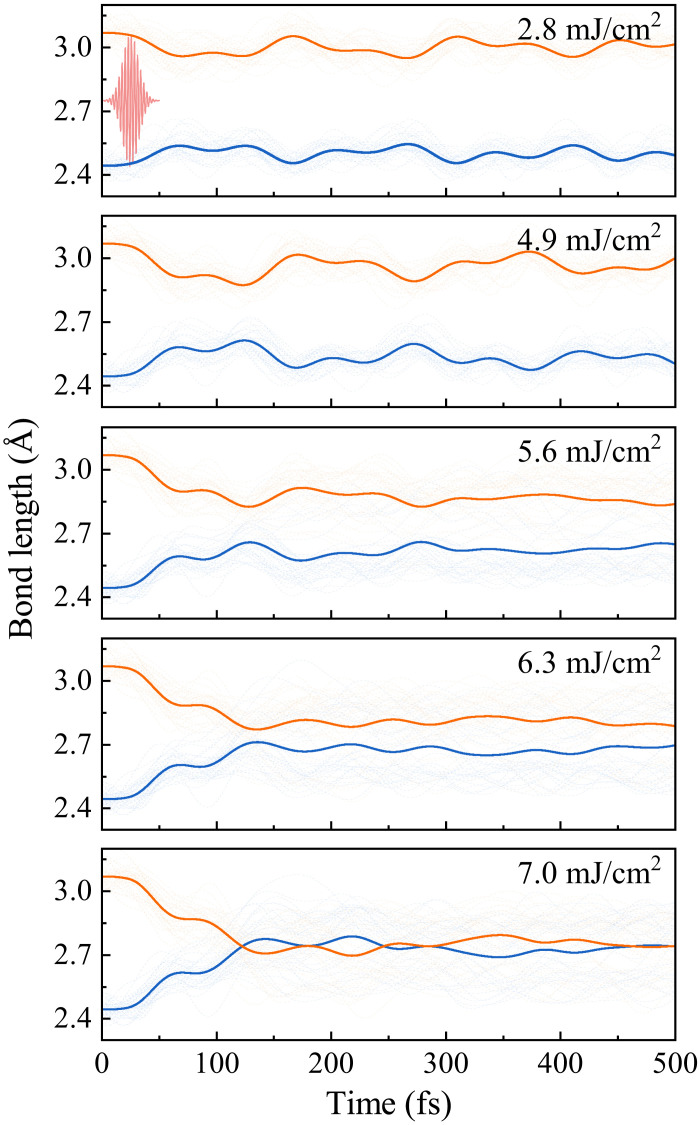
Evolution of V-V bond lengths under varying pump fluences (2.8, 4.9, 5.6, 6.3, and 7.0 mJ/cm^2^). Light blue and light orange dashed lines represent individual short and long V-V bonds, respectively. Solid blue and orange lines denote the average lengths of short and long bonds.

VO_2_ is a prototypical phase-change material that exhibits both thermally driven (*16*–*18*) and optically driven phase transitions (*19*–*22*). At low temperatures, VO_2_ crystallizes in a low-symmetry monoclinic (M1) phase, characterized by alternating short and long V-V bonds along the rutile *c* axis (Fig. 1A). The short V-V bonds form dimers, leading to a splitting of the *a_1g_* orbital into bonding $d_{\|}$ ($d_{x^{2}-y^{2}}$) and antibonding $d_{\|}^{*}$ states (*23*). This orbital splitting opens a bandgap, resulting in an insulating state (Fig. 1A). As the temperature rises above ~340 K, VO_2_ undergoes a transition to the high-symmetry rutile (R) phase, where the V-V dimers dissolve, the alternation in V-V bond lengths disappears, and the $d_{\|}$ bonding and antibonding states merge. This band merging closes the gap, yielding a metallic phase (*16*, *17*). Numerous ultrafast experiments have demonstrated that a single femtosecond near-infrared laser pulse above a threshold fluence can drive the M1-to-R phase transition (*19*–*22*, *24*–*28*). Our previous work (*23*, *29*) has revealed that this photoinduced dissociation of V-V dimers in VO_2_ is driven by the atomic driving force arising from the occupation of photoexcited holes on V-V dimerized bonding states ($d_{\|}$). As shown in Fig. 1A, in our simulation, a 50-fs, 800-nm laser pulse with a fluence of 7 mJ/cm^2^ excites sufficient electrons from the $d_{\|}$ bonding state to the conduction bands in the M1 phase, leaving photoexcited holes in the $d_{\|}$ bonding state. This generates enough driving force to break all V-V dimers (Fig. 2), thereby inducing a structural transition to the R phase, accompanied by an insulator-to-metal transition.

As the laser fluence is slightly reduced below the threshold, the advanced transient diffuse x-ray scattering experiment (*13*) and our recent work (*29*) reveal the emergence of localized structural transitions originating from the self-trapping process of photoexcited holes. This self-trapping process leads to the formation of a metastable disordered phase, as the driving forces are insufficient to break all V-V dimers at fluences of 5.6 and 6.3 mJ/cm^2^ (Fig. 2). At these laser fluences, in addition to such localized disordered atoms, the remaining atoms exhibit collective ~6-THz oscillations (fig. S2), indicating the coexistence of localized disorder and coherent motion. This behavior is supported by experiments, which observe the evolution of long-range ordered Bragg peaks associated with a ~6-THz phonon, along with the substantial enhancement of diffuse scattering between Bragg peaks indicative of disorder (*13*). When the fluence is further decreased below 4.9 mJ/cm^2^, none of the V-V dimers are broken, and the V-V dimer lengths instead exhibit pronounced ~6-THz coherent oscillations (Fig. 2 and fig. S1). These oscillations are consistent with coherent phonon modes observed experimentally during PIPTs in VO_2_ (*22*, *26*, *30*, *31*). In addition, we identify a higher-frequency ~14-THz phonon mode superimposed on the dominant ~6-THz oscillation (fig. S3), which originates from V-V bond stretching along the *x* direction. This oscillation does not directly affect the overall structural transition, but it modulates the periodic evolution of the bandgap at low laser fluence.

Here, our simulations indicate a continuous evolution from a regime dominated by coherent phonons at low fluence, to a coexistence of localized disorder and coherent motion at intermediate fluence, and lastly to fully disordered transitions near the threshold fluence. We find that by selectively activating and controlling these two distinct dynamic regimes—coherent oscillations at low fluence and incoherent local transitions near the threshold—we can probe the distinct electron and phonon dynamics, simultaneously enhancing the switching efficiency of the system using tailored double-pulse excitation.

### Coherent control of PIPTs via time-delay in two-pulse excitation

**Fig. 3.**
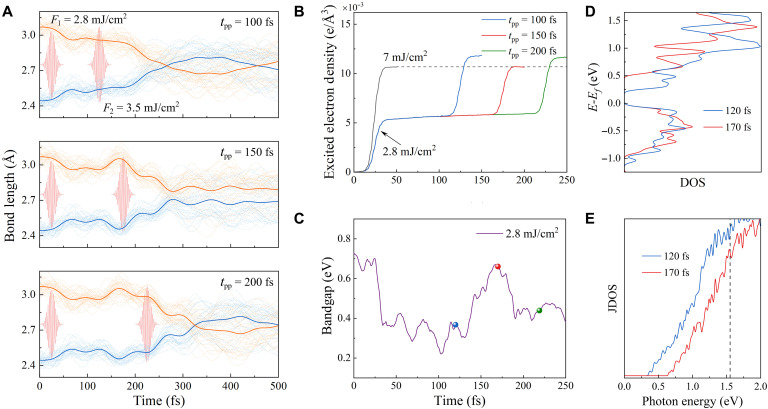
Coherent control of PIPTs via two-pulse excitation. (**A**) Time evolution of V-V bond lengths under a first 50-fs pump pulse (2.8 mJ/cm^2^, 800 nm), followed by a second 50-fs pulse (3.5 mJ/cm^2^, 800 nm) applied at delay times of 100, 150, and 200 fs. Light blue and light orange dashed lines denote individual short and long V-V bonds, respectively, while solid blue and orange lines represent their corresponding average lengths. (**B**) Number of photoexcited electrons transferred from the valence bands to the conduction bands under various pump conditions, including a single 50-fs pulse (7.0 mJ/cm^2^) and double-pulse excitation consisting of a first 50-fs pulse (2.8 mJ/cm^2^) followed by a second 50-fs pulse (3.5 mJ/cm^2^) at delay times of 100, 150, and 200 fs. (**C**) Temporal evolution of the bandgap under a single-pulse excitation at 2.8 mJ/cm^2^. The small spheres represent the bandgap magnitude at the moment when the second pulse reaches its peak intensity. (**D**) Time-dependent density of states (TDDOS) at a single pump fluence of 2.8 mJ/cm^2^. The blue and red lines correspond to the TDDOS at 120 fs (smaller gap, ≈ 0.3 eV) and at 170 fs (larger gap, ≈ 0.6 eV), respectively. (**E**) The corresponding joint density of states (JDOS) at 120 fs (blue line) and 170 fs (red line).

According to the above understanding of single-pulse dynamics, we next examine how the application of a second pulse at various time delays affects the following dynamic process and transition efficiency in the presence of coherent structural oscillations initiated by an initial low-fluence pulse (~2.8 mJ/cm^2^, 800 nm). Figure 3A shows the time evolution of V-V bond lengths within the first 500 fs under double-pulse excitation, where the second pulse (3.5 mJ/cm^2^, 800 nm) is applied with delay times of 100, 150, and 200 fs, defined as the peak-to-peak separation between the two pulses. The corresponding effective excitation window of the second pulse (shaded regions in fig. S4) is a time range that covers both the minimum and maximum of the ~6-THz oscillation (e.g., around 125 and 167 fs). When the second pulse is applied at 100 or 200 fs, all V-V dimers are broken at ~260 and 320 fs, respectively, completing the structural transition from M1 phase to R phase, in which the long and short V-V bonds become equivalent and the corresponding bandgap collapses (fig. S5, A and C). Notably, the total fluence required for the transition in these cases is only 6.3 mJ/cm^2^, which is lower than the single-pulse threshold of 7.0 mJ/cm^2^. On the basis of the energy efficiency metric defined in previous studies (*13*) as $\eta =\frac{F_{\text{th}}^{\text{single}}-F_{\text{th}}^{\text{double}}}{F_{\text{th}}^{\text{single}}}$, this corresponds to an enhancement of ~10%. The 10% energy saving only reflects the internal energy associated with the light fluence absorbed by the material. From an application point of view, one might also need to consider the change of light absorption and reflect at the sample surface due to the electron and phonon excitation, which are beyond the scope of current study. In contrast, when the second pulse is applied at a 150-fs delay, the short V-V bonds show substantial elongation and the long bonds notable shortening around 270 fs, accompanied by local phase transitions that induce a bandgap collapse (fig. S5B). However, the bond lengths do not fully converge, indicating an incomplete phase transition with a weak metallic property.

This variation in energy efficiency arises from the interplay between coherent structural dynamics and the resulting modulation of the electronic structure. This variation in energy efficiency can be attributed to the underlying electronic response governed by coherent structural dynamics. Periodic modulation of electronic states by coherent lattice vibrations has been experimentally observed in another transition metal oxides (*32*). In our case, as shown in Fig. 3C, the bandgap evolution following the initial 2.8 mJ/cm^2^ pulse exhibits periodic oscillations, driven by coherent V-V dimer motion. Specifically, elongation of V-V dimers raises the energy of the bonding $d_{\|}$ states and lowers that of the antibonding $d_{\|}^{*}$ states, while contraction has the opposite effect (*33*). This results in a time-dependent bandgap oscillation (Fig. 3C), which can be directly probed by time- and angle-resolved photoemission spectroscopy (*34*, *35*). When the second pulse arrives near a bandgap minimum, such as at ~100 or ~200 fs, it efficiently promotes a larger number of electrons on $d_{\|}$ states into the conduction bands, surpassing the carrier density threshold for a full phase transition even with a total fluence of only 6.3 mJ/cm^2^ (Fig. 3B). In contrast, a second pulse delayed by ~150 fs, although still producing more excited carriers than a single pulse of equal fluence (fig. S7), fails to reach the critical excitation density. This is attributed to the larger instantaneous bandgap at 150 to 180 fs, which reduces the joint density of states at the photon energy of 1.55 eV compared with that at 100 to 130 fs (Fig. 3E and fig. S9). As a result, fewer electronic states satisfy the optical transition condition (Fig. 3D and fig. S8), reducing the number of electrons promoted to the conduction band and preventing a complete structural transition. Therefore, the periodic modulation of the bandgap governs the timing-dependent efficiency of photoexcitation, leading to an oscillatory dependence of transition yield on the delay time of the second pulse.

To characterize the oscillatory behavior of bond length and bandgap at different temperatures (fig. S10), we use laser fluences of 7.7, 5.6, and 4.3 mJ/cm^2^ for 50, 100, and 300 K, respectively, each chosen to be below the corresponding threshold fluence. Since the phase-transition threshold in VO_2_ decreases with increasing initial temperature (*26*, *36*), a lower laser fluence is used at higher temperatures. The synchronization between bond length and bandgap remains consistent across these temperatures, demonstrating that our theoretical framework is applicable at various of temperatures. These mechanistic insights may help explain experimentally observed delay time-dependent modulation of phase transition efficiency in systems such as In-Si nanowires (*14*) and 1T-TaS_2_ (*15*).

Furthermore, to explore the dependence of bandgap modulation on the photon energy of the second pulse, we performed additional dynamical simulations using 1050-nm (1. 18 eV) and 1600-nm (0.78 eV) pulses (fig. S13). We found that the higher-energy 1050-nm pulse exhibits behavior similar to that of the previously used 800-nm pulse. In contrast, the lower-energy 1600-nm pulse is much less effective in promoting the phase transition, due to the presence of a bandgap that prevents excitation of the $d_{\|}$ orbitals over the entire energy range (figs. S14 to S16). The resulting driving force is therefore insufficient to induce a complete structural transition. These results suggest that, in these cases, the photon energy should be chosen to exceed the bandgap sufficiently to allow excitation of all $d_{\|}$ orbitals involved in the transition.

To further verify the phase effect between pulses, we performed additional simulations using two pulses with a relative phase difference of π/2 and a delay of 100 fs (fig. S17, A and C). The results are nearly identical to those without phase variation (fig. S17B), indicating that electronic interference effects are negligible on the timescale relevant to the phase transition, because the delay between the two pump pulses of 100 to 200 fs is much longer than the subcycle timescale of electronic interference (~2 to 3 fs for 800-nm light). Ultrafast electronic interference on subcycle timescales induced by phase-locked double pulses (*37*–*40*) remains an interesting direction for future investigation in attosecond science.

### Incoherent localized dynamics as a control knob for PIPTs via two pump pulses

**Fig. 4.**
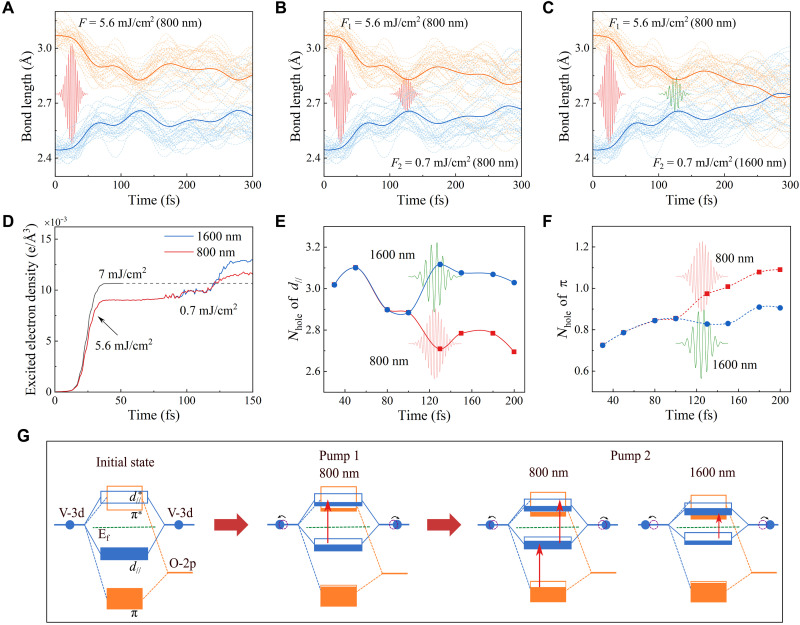
Controlling of incoherent localized dynamics in PIPTs via two pump pulses. (**A**) Time evolution of V-V bond lengths under a single 50-fs pump pulse (5.6 mJ/cm^2^, 800 nm). (**B** and **C**) Same as (A), but with an additional second 50-fs pump pulse (0.7 mJ/cm^2^) applied at a delay of 100 fs, using 800 nm in (B) and 1600 nm in (C). (**D**) Number of photoexcited electrons under various pump conditions, including a single 50-fs pulse (7.0 mJ/cm^2^) and double-pulse excitation with a first 50-fs pulse (5.6 mJ/cm^2^) followed by a second 50-fs pulse (0.7 mJ/cm^2^) at 100-fs delay. (**E** and **F**) Time evolution of photoexcited hole populations in the $d_{\|}$ orbital (E) and π orbital (F) under second-pump excitation at 800 or 1600 nm. (**G**) Schematic illustration of the evolution of photoexcited electrons under second-pump excitation at 800 or 1600 nm. Lower photon energy (1600 nm) more effectively excites the electrons of $d_{\|}$ bonding states.

On the other hand, when the fluence of the first pulse is increased to 5.6 mJ/cm^2^ (800 nm), some local V-V dimers are broken around ~80 fs, driven by the self-trapping of photoexcited holes (*29*), while the overall structure still retains the characteristic contrast between short and long bonds (Fig. 4A). This result is indicative of a local structural phase transition (*29*) or the formation of photoinduced polarons (*13*, *41*). These photoinduced localized structural transitions lead to a bandgap collapse within ~100 fs, rather than exhibiting coherent oscillations (fig. S6A), and precede the completion of the M1-to-R phase transition (*36*). This behavior reflects a coexistence of disordered and coherent motion, rather than purely coherent dynamics characterized by bandgap oscillations (Fig. 3C). Such mixed dynamics weaken the overall coherent response and may explain the absence of oscillations in the threshold energy observed experimentally (*13*). Once the first pulse induces localized polarons, the wavelength of the second pulse becomes the determining factor for the efficiency of the completely structural phase transition. As shown in Fig. 4B, when a weaker 800-nm second pulse (0.7 mJ/cm^2^) is applied at a delay time of 100 fs, the V-V bond lengths exhibit negligible changes compared to the single-pulse case (Fig. 4, A and B). In contrast, replacing the second pulse with a longer-wavelength 1600-nm pulse (0.7 mJ/cm^2^) causes the V-V bond lengths to evolve toward global equality at ~280 fs (Fig. 4C), indicating more effective dimer dissociation and the realization of a complete phase transition. Notably, this also results in an approximate 10% improvement in energy efficiency relative to single-pulse excitation.

To understand the underlying mechanism, we examine the number of photoexcited electrons under these conditions (Fig. 4D). Unexpectedly, both double-pulse scenarios generate carrier densities that exceed the transition threshold fluence of 7 mJ/cm^2^ established for single-pulse excitation. This observation suggests that the total number of photoexcited electrons alone is not a sufficient predictor of phase transition efficiency. Instead, the detailed nature of the atomic driving force behind PIPTs in VO_2_ must be considered. Previous studies have shown that photoexcited electrons originating from the $d_{\|}$ bonding states, which leave behind photoexcited holes, are the key driving force for V-V dimer dissociation (*23*, *29*). In contrast, the lower-energy π bonding states formed by the interaction between V-3d and O-2p orbitals have minimal impact on V-V bond breaking (Fig. 4G, initial state), as these states are oriented nearly perpendicular to the V-V bonding direction (*23*).

To quantitatively analyze orbital-specific electronic excitations during photoexcitation, we examine the electronic time-dependent density of states shown in fig. S18. It illustrates the evolution of photoexcited electrons (in red) and holes (in blue) over time. We integrate the distributions of photoinduced holes over the energy ranges of −1.5 to 0 eV (relative to the Fermi level) and −3.5 to −2 eV to estimate the number of holes originating from the $d_{\|}$ and π bonding states, respectively (Fig. 4, E and F). As shown in Fig. 4E, the 800-nm second pulse reduces the population of photoexcited holes on the $d_{\|}$ orbitals around the end of the second pulse (∼150 fs). Simultaneously, it preferentially excites electrons from the deeper-lying π bonding states, thereby generating more photoexcited holes in the π bonding states (Fig. 4F), which are less involved in driving the structural phase transition. This behavior can be attributed to the fact that the electrons of $d_{\|}$ states have already been substantially depleted by the stronger first pulse (Fig. 4G), making further excitation of these states less favorable due to a band-filling effect and a Pauli exclusion principle among photoexcited carriers (*42*). As a result, the less-excited π states become the primary excitation channels for the 800-nm second pulse with higher photon energy (Fig. 4G), leading to a reduction in effective holes in the $d_{\|}$ states, which in turn decreases the formation of local polarons and hinders the development of a global phase transition (Fig. 4B). In contrast, the second 1600-nm pulse, with lower photon energy, selectively excites the $d_{\|}$ states that directly contribute to V-V dimer dissociation while effectively avoiding excitation of the deeper-lying π states (Fig. 4, E to G). Ultimately, more local V-V dimers are dissociated, leading to a complete structural phase transition (Fig. 4C). Notably, unlike the presence of band-filling effect at higher excitation, the number of electrons excited from the $d_{\|}$ states to the conduction bands remains very small at lower fluences (2.8 mJ/cm^2^) in the above-discussed coherent case, so that the second pulse still predominantly excites electrons from the $d_{\|}$ orbitals and the contribution from the π orbitals can be safely neglected due to the very small number of empty $d_{\|}$ states (fig. S16).

To further confirm the suitable wavelength range of the second pulse that avoids excitation from the lower π state to the $d_{\|}$ state, additional simulations were performed at 1240 nm (1.00 eV) and 1050 nm (1.18 eV) (fig. S19) under an incoherent case. We found that when the second pulse wavelength is ≥1240 nm, it can efficiently trigger the global phase transition. This is consistent with the energy gap between the π orbital and $d_{\|}$ orbital, which is ~1 eV (fig. S19). Therefore, on the basis of our theoretical simulations and analysis, we propose that in future multipulse PIPTs experiments in solids, if the initially formed localized polarons sufficiently modify the electronic density of states, then subsequent pulses with carefully selected wavelengths can selectively excite electronic states that are directly involved in the structural transition, thereby enhancing the overall phase transformation efficiency (*13*–*15*, *43*–*45*). Furthermore, we also propose a possible strategy to control the formation and annihilation of polarons through wavelength-selective photoexcitation of different orbital transitions, which is crucial for controlling carrier transport in metal oxides and other materials (*46*–*50*). However, at lower temperatures (e.g., 100 K), the localized phase transition induced by the first pulse becomes difficult to realize (fig. S11), because weaker local atomic thermal oscillations are unfavorable for the self-trapping of photoexcited holes (*51*), leading instead to collectively coherent motion. In contrast, in other systems with higher transition temperatures, excessive lattice thermal fluctuations (>600 K) can strongly perturb the photoinduced polarons, leading to their rapid decay, as in NbO_2_ (*51*). Therefore, both insufficient and excessive lattice fluctuations are unfavorable for the formation of photoinduced polarons, particularly in materials with much higher transition temperatures. These results indicate that the efficiency modulation by localized polarons is strongly temperature dependent.

## DISCUSSION

In summary, we have used rt-TDDFT to systematically investigate the microscopic mechanisms of PIPTs in VO_2_ under double-pulse laser excitations. Our results reveal that the fluence of the first pulse governs the nature of the initial structural response, either inducing coherent lattice oscillations or triggering localized, incoherent transitions. In both cases, a properly tuned second pulse can enhance the overall transition efficiency by ~10%. By introducing the concept of effective electron excitation, we establish a mechanistic link between structural dynamics, orbital-selective excitation, and energy-efficient phase switching. Under weak initial excitation, the first pulse induces a collective lattice motion that periodically modulates the band structure, resulting in delay time-dependent variations in carrier excitation. Conversely, under stronger subthreshold excitation, incoherent local transitions substantially modify the electronic structure. In this regime, wavelength-optimized pulses can selectively excite the $d_{\|}$ bonding states that drive the V-V dimer dissociation, facilitating the evolution from local to global phase transformation. Our study shows that the multipulse method can probe and control the complex ultrafast dynamics of the laser-induced phonon and electron excitation. The first pulse induces different excitations, and the subsequent time evolution of electronic and lattice behaviors can be revealed by the second pulse (the time delay dependence can reveal the lattice dynamics, and wavelength dependence can reveal the electronic structure change). The photoinduced polaron–assisted mechanism may also occur in other correlated materials where strong coupling between photoexcited carriers and lattice distortions leads to carrier localization, as proposed in our recent study of NbO_2_ (*51*) and in related systems such as Fe_2_O_3_ (*47*, *52*) and WO_3_ (*53*). We believe that the multipulse method offers an effective approach to probing and controlling polaron-assisted dynamics in these correlated materials.

## MATERIALS AND METHODS

All calculations were performed using the PWmat software package (*54*). The local density approximation functional with a Hubbard U correction of 3.4 eV (*55*) was used for the exchange-correlation, yielding a bandgap of ~0.7 eV in VO_2_, consistent with experimental values (*24*, *56*). In our previous work on PIPTs in VO_2_, we verified that varying the *U* value does not alter the essential evolution behavior of the atomic and electronic structures (*29*). Hence, the influence of *U* variations is neglected in the present study. A plane-wave basis set with a cutoff energy of 50 rydberg was used. Simulations were carried out on a 216-atom supercell with a 1 × 1 × 1 Monkhorst-Pack k-point mesh.

The photoexcited structural and electronic dynamics were simulated using rt-TDDFT, implemented in PWmat under the Ehrenfest dynamic framework (*57*, *58*), which self-consistently accounts for real-time electron-ion coupling (see section S1 for details). In our simulations, we used a time step of 0.1 fs by time-dependent linear Hamiltonian approximation (*57*) and initiated structural configuration from a Born-Oppenheimer molecular dynamics equilibrated at corresponding temperatures. To better reflect experimental conditions, all simulations reported here are performed at 300 K. To mimic photoexcitation, we use an external electric field (section S1) with a Gaussian shape

$$E\left(t\right)=E_{0}\text{cos}\left(\omega t\right)\text{exp}\left[-{\left(t-t_{0}\right)}^{2}/\left(2\sigma^{2}\right)\right]$$ (1)

For the first pulse, we used a photon energy of ω = 1.55 eV (800 nm) (*24*, *27*), a central time delay of $t_{0}$ = 25 fs, and a width parameter $\sqrt{2}\sigma$ = 10 fs, corresponding to a full width at half maximum of 16 fs. The second pulse had either ω (800 nm) or 0.78 eV (1600 nm), with time delay $t_{0}$ = 125, 175, or 225 fs. The electric field *E*_0_ is adjusted to attain different electronic excitations at different laser fluences (fig. S7). The electric field is applied along the [111] direction of VO_2_ and treated as spatially uniform within the simulation cell along the *x*, *y*, and *z* axes, making the results largely insensitive to polarization (*23*, *29*). In addition, a 35-fs laser pulse is used, consistent with the experimental conditions (*13*), and the photoinduced structural dynamics remain unchanged (fig. S20).

To assess the effect of supercell size on localized polarons, we performed simulations with 96-atom and 216-atom systems. Approximately 6% of the V-V dimers are broken in the 96-atom supercell, compared to about 6 to 8% in the 216-atom case (fig. S12), indicating a proportional increase with system size. Therefore, in our study, the finite-size effect on photoexcited polarons is negligible.

## Supplementary Materials

### This PDF file includes:

Sections S1 to S5 Figs. S1 to S20
