# Supplementary material for: Probing and controlling coherent and incoherent dynamics of phase transitions via multipulse excitation
Source: Sci Adv. 2026 Jun 17;12(25):eaef8585. doi: 10.1126/sciadv.aef8585 (PMC13274594; doi:10.1126/sciadv.aef8585)
Supplement: Supplementary file 1 — Sections S1 to S5 Figs. S1 to S20 [file sciadv.aef8585_sm.pdf]

Science Advances
AAAS

Supplementary Materials for

Probing and controlling coherent and incoherent dynamics of phase transitions via multipulse excitation

Feng-Wu Guo *et al.*

Corresponding author: Wen-Hao Liu, liuwh@semi.ac.cn; Lin-Wang Wang, lwwang@semi.ac.cn; Jun-Wei Luo, jwluo@semi.ac.cn

Sci. Adv. **12**, eaef8585 (2026)
DOI: 10.1126/sciadv.aef8585

This PDF file includes:

Sections S1 to S5
Figs. S1 to S20

Section 1: Theory and methodology

Within the rt-TDDFT algorithm (*57, 58*), the time-dependent Kohn-Sham equation is:

$$i\frac{\partial \varphi_i(t)}{\partial t} = H[\rho(t)]\varphi_i \quad \text{(S1)}$$

The time-dependent wave functions are expanded by the instantaneous adiabatic eigenstates.

$$\varphi_i(t) = \sum_l C_{i,l}(t)\,\phi_l(t), \quad H(t)\phi_l(t) \equiv \varepsilon_l(t)\phi_l(t) \quad \text{(S2)}$$

The coefficients $C_{i,l}(t)$ are calculated according to Eq. S2:

$$\dot{C}_{i,l}(t) = -i\varepsilon_l(t)C_{i,l}(t) - \sum_k C_{i,k}(t)V_{lk}(t) \quad \text{(S3)}$$

where $V_{lk}(t) = \langle \phi_l(t)|\partial\phi_k(t)/\partial t\rangle$ represents the nonadiabatic couplings. To evaluate these terms, the Hamiltonian is approximated by linear interpolation during each time step (*57*).

$$H(t) = H(t_1) + \frac{t - t_1}{(t_2 - t_1)} \times \left(H(t_2) - H(t_1)\right) \quad \text{(S4)}$$

which allows stable propagation with larger time steps up to 0.1-0.5 fs without loss of accuracy.

Photoexcitation is simulated by introducing the vector potential **A**(t) into the Hamiltonian,

$$i\hbar\frac{\partial}{\partial t}\varphi_i(\mathbf{r},t) = \left\{\frac{(\mathbf{p} - \frac{e}{c}\mathbf{A}(t))^2}{2m} + \int d\mathbf{r}' e^{\frac{ie}{\hbar c}\mathbf{A}(t)\cdot\mathbf{r}} V_{ion}(\mathbf{r},\mathbf{r}')\, e^{-\frac{ie}{\hbar c}\mathbf{A}(t)\cdot\mathbf{r}'} + V_H[n] + V_{\mathrm{xc}}[n]\right\}\varphi_i(\mathbf{r},t) \quad \text{(S5)}$$

with the electric field defined by

$$\boldsymbol{E}(t) = -\frac{1}{c}\frac{\partial \boldsymbol{A}(t)}{\partial t}, \qquad \boldsymbol{E}(t) = E_0\, cos(\omega t)\, exp[-(t - t_0)^2/(2\sigma^2)] \quad \text{(S6)}$$

The atomic forces and nuclear dynamics are described by Ehrenfest dynamics:

$$M_I\frac{d^2\boldsymbol{R}_I(t)}{dt^2} = \boldsymbol{F}_{R_I}(t), \qquad \boldsymbol{F}_{R_I} = \sum_l \langle\varphi_l|\nabla_{R_I}H(\rho(t))|\varphi_l\rangle \quad \text{(S7)}$$

In this framework, since the time-dependent Hamiltonian depends explicitly on the instantaneous ionic positions $\boldsymbol{R}(t)$, the motion of ions continuously alters the electronic potential energy surface, and the resulting change in the electronic density exerts back forces on the ions. This mutual dependence enables a real-time, fully microscopic description of electron-phonon coupling, allowing direct energy exchange between electrons and ions.

Section 2: Analysis of phonon modes

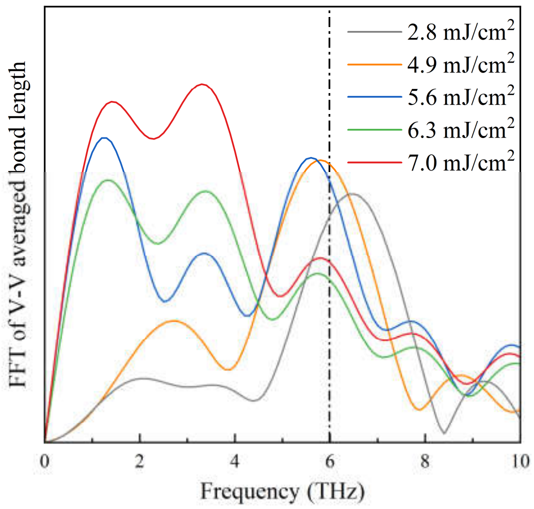

Fig. S1. Coherent phonon modes. The coherent phonon modes are obtained by performing a fast Fourier transform (FFT) on the dynamic evolution of the averaged bond length of V-V dimers following the single pulse photoexcitation in Fig. 2.

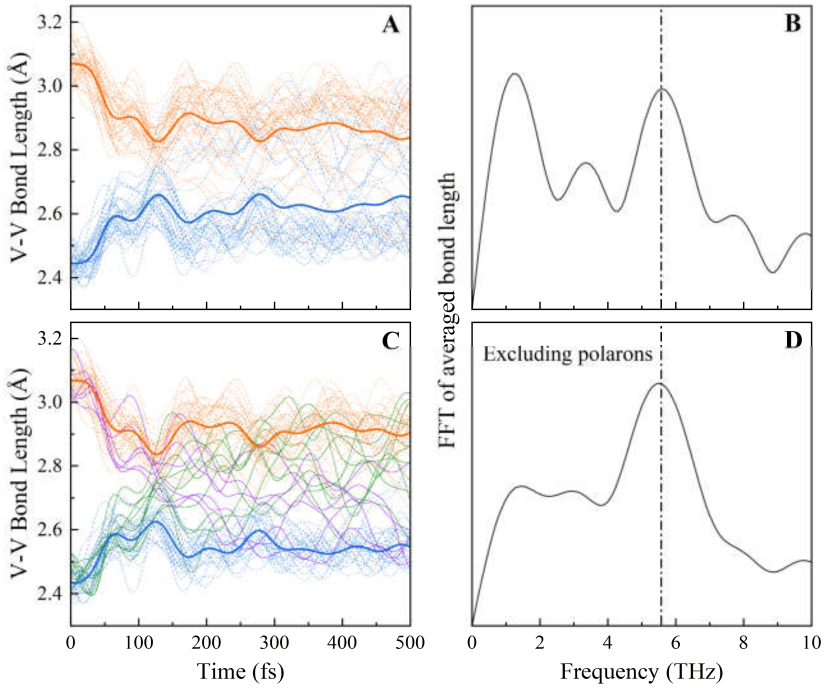

Fig. S2. Coherent phonon modes under laser fluence of 5.6 mJ/cm^2. (A, B) Evolution of V-V bond lengths under laser fluence of 5.6 mJ/cm^2, and corresponding coherent phonon modes obtained by performing a fast Fourier transform (FFT) of the time evolution of the averaged V-V bond lengths. Light blue and light orange dashed lines represent individual short and long V-V bonds, respectively, while the solid blue and orange lines denote their averaged values. **(C, D)** Same as **(A, B)**, but excluding bonds

involved in localized structural transitions when performing the FFT; these bonds are labeled by green and purple lines.

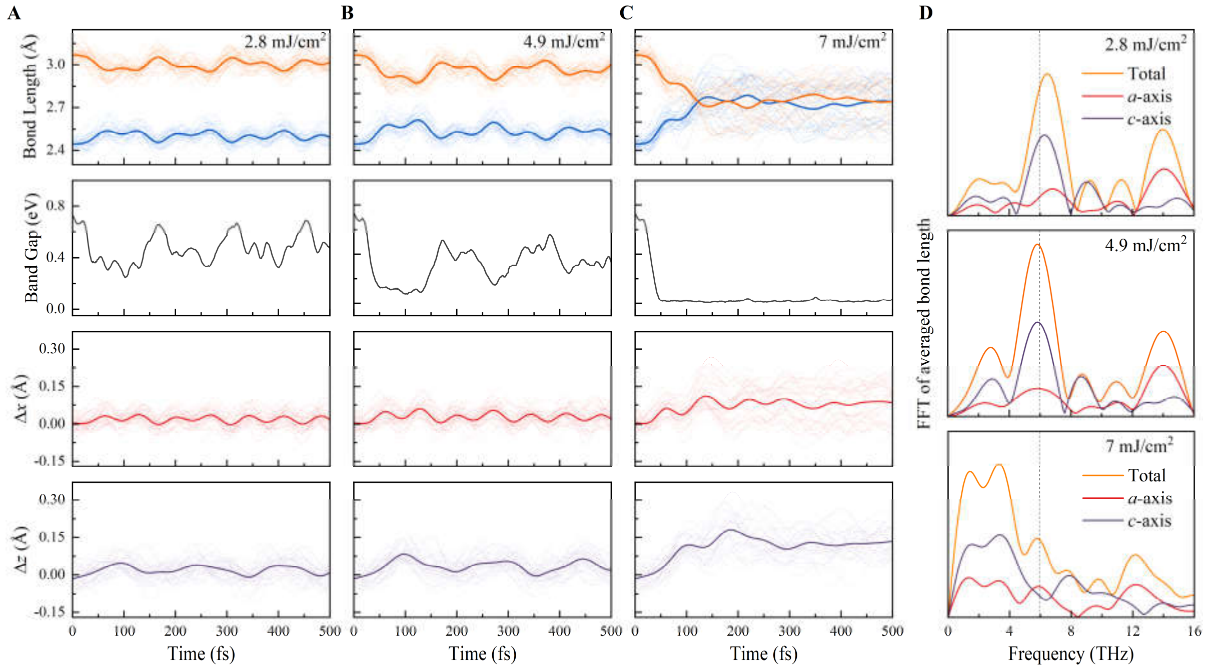

Fig. S3. Coherent phonons and V-V bond dynamics along *x* and *z* axes. (A-C) Time evolution of the V-V bond lengths, bandgaps and the displacements of V atoms along the *x* and *z* axes at fluences of 2.8 mJ/cm^2, 4.9 mJ/cm^2 and 7 mJ/cm^2, respectively. **(D)** The corresponding phonon modes obtained from the Fourier transform (FFT).

As shown in Fig. S3, the additional 14 THz mode originates from V-V bond stretching along the *a*-axis, while such motion is absent along the *c*-axis. The ~6 THz phonon at 2.8 and 4.9 mJ/cm^2 primarily arise from motions along the *z*-axis (Fig. S3C). At the structural transition threshold fluence of 7.0 mJ/cm^2, the system predominantly exhibits a broad spectrum of disordered motions in the 1-5 THz range. Correspondingly, the atomic displacements along both the *x*-axis and *z*-axis become increasingly disordered. Along the *x*-axis, weak residual coherent phonon components at ~6 THz and ~12 THz persist, reflecting irregular, small-amplitude oscillations along the *x*-axis (Fig. S4). This oscillation does not directly affect the overall structural transition, but it modulates the periodic evolution of the band gap (Fig. S3). By exploiting this modulation of the gap, a second laser pulse can be used to control electron excitation and thereby accelerate the structural phase transition.

Section 3: Evolution of photoexcited carriers, atomic structure, and electronic structure

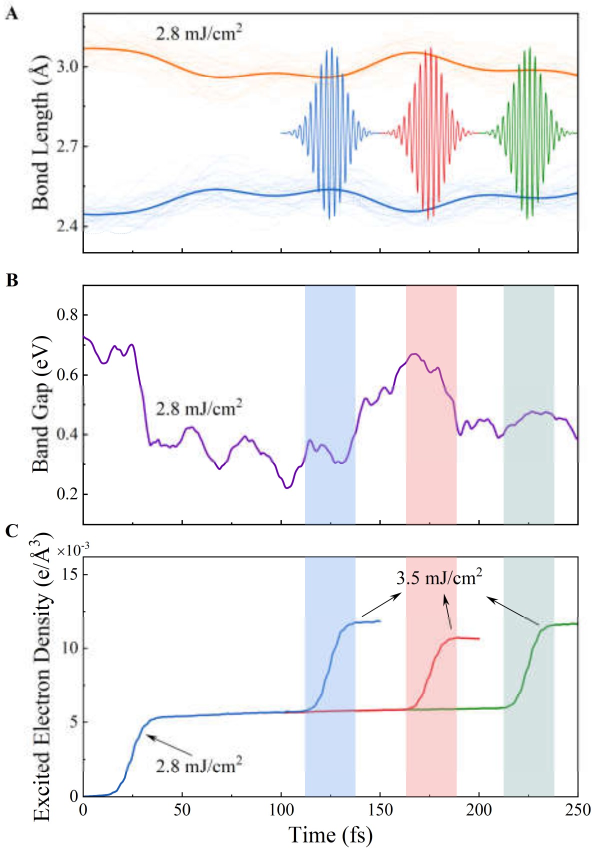

Fig. S4. Time-resolved double-pulse waveform and effective excitation window. (A) Time evolution of the V-V bond lengths at a fluence of 2.8 mJ/cm^2 induced by the first pulse. The blue, red, and green lines indicate the applying time of the second pulse. **(B)** Temporal evolution of the bandgap under a fluence of 2.8 mJ/cm^2 induced by the first pulse. **(C)** Number of photoexcited electrons under double-pulse excitation, consisting of a first 50 fs pulse (2.8 mJ/cm^2) followed by a second 50 fs pulse (3.5 mJ/cm^2) with delay times of 100 fs, 150 fs, and 200 fs. The blue, red, and green shaded areas in **(B-C)** denote the effective pump window of the second pulse.

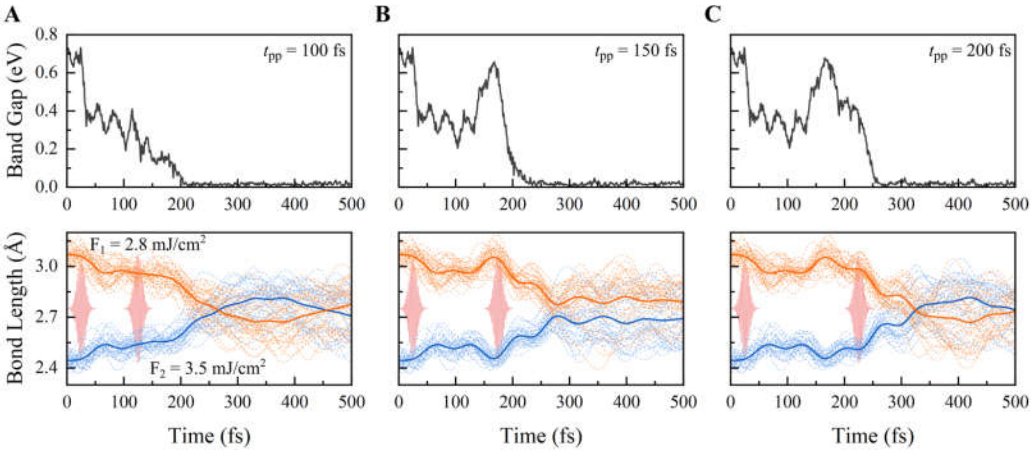

Fig. S5. Bandgap and V-V bond length dynamics under coherent control conditions. (A-C) Time evolution of the band gap and V-V bond lengths under a first 50-fs pump pulse (2.8 mJ/cm^2, 800 nm), followed by a second 50-fs pulse (3.5 mJ/cm^2, 800 nm) applied at delay times of 100 fs, 150 fs, and 200 fs. Light blue and light orange dashed lines denote individual short and long V-V bonds, respectively, while solid blue and orange lines represent their corresponding average lengths.

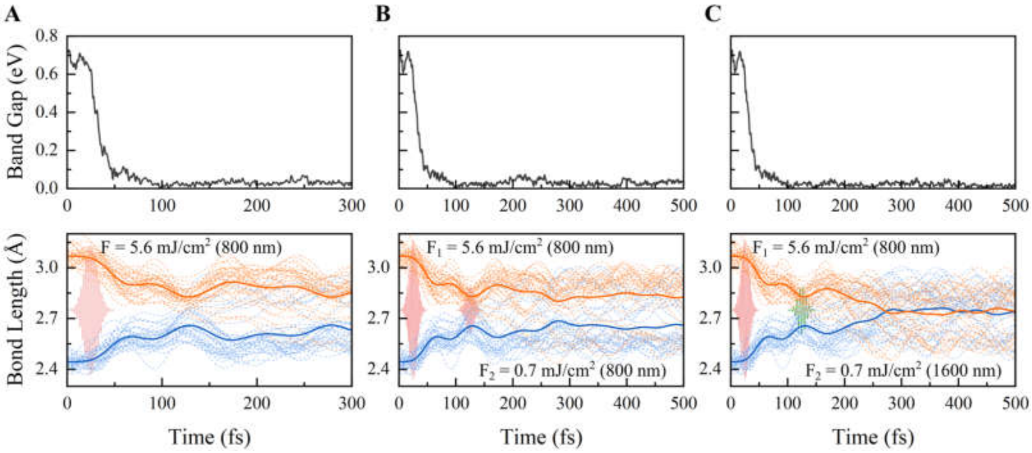

Fig. S6. Bandgap and V-V bond length dynamics under local control conditions. (A) Time evolution the band gap of V-V bond lengths under a single 50-fs pump pulse (5.6 mJ/cm^2, 800 nm). **(B, C)** Same as **(A)**, but with an additional second 50-fs pump pulse (0.7 mJ/cm^2) applied at a delay of 100 fs, using 800 nm in **(B)** and 1600 nm in **(C)**.

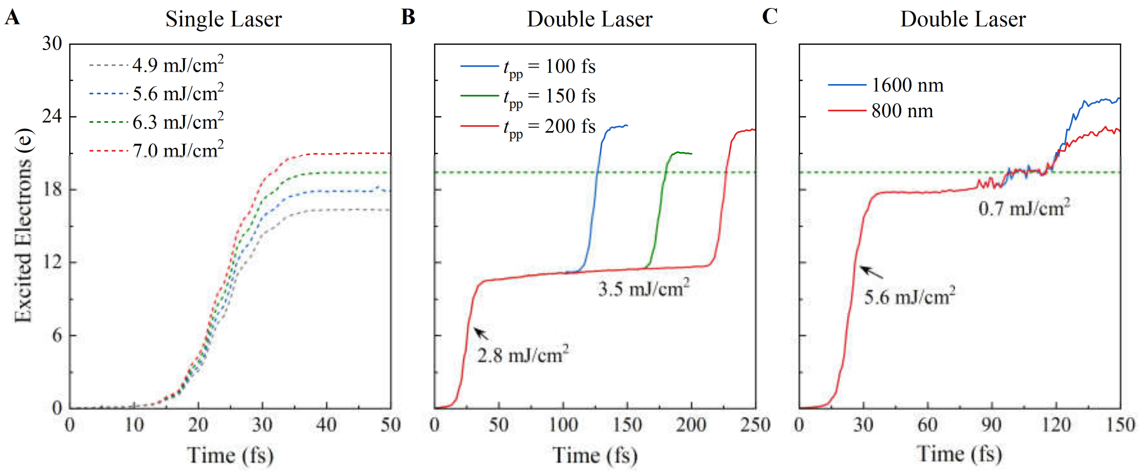

Fig. S7. The number of photoexcited electrons transferred from the valence band to the conduction band under different pump conditions. (A) Single 50-fs pump pulse at fluences of 4.9, 5.6, 6.3, and 7.0 mJ/cm^2. **(B)** Double-pulse excitation: the first 50-fs pulse is 2.8 mJ/cm^2, followed by a second 50-fs pulse of 3.5 mJ/cm^2 applied at delay times of 100 fs, 150 fs, and 200 fs. **(C)** Double-pulse excitation: the first 50-fs pulse is 5.6 mJ/cm^2, followed by a second 50-fs pulse of 0.7 mJ/cm^2 (800 nm or 1600 nm) at a delay of 100 fs.

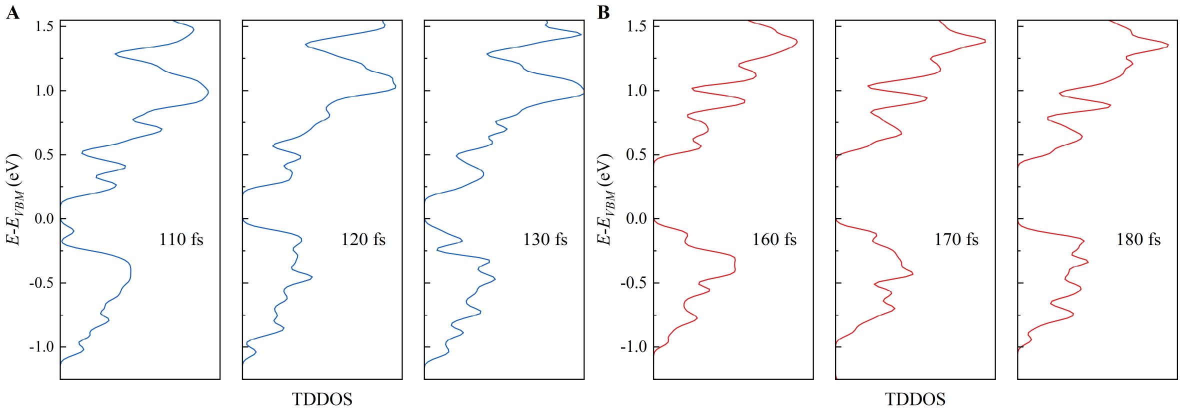

Fig. S8. Time-dependent density of states (TDDOS). (A-B) TDDOS at a single pump fluence of 2.8 mJ/cm^2. The blue and red lines correspond to the TDDOS at 110, 120, and 130 fs (smaller gap, ≈ 0.3 eV) and at 160, 170, and 180 fs (larger gap, ≈ 0.6 eV), respectively.

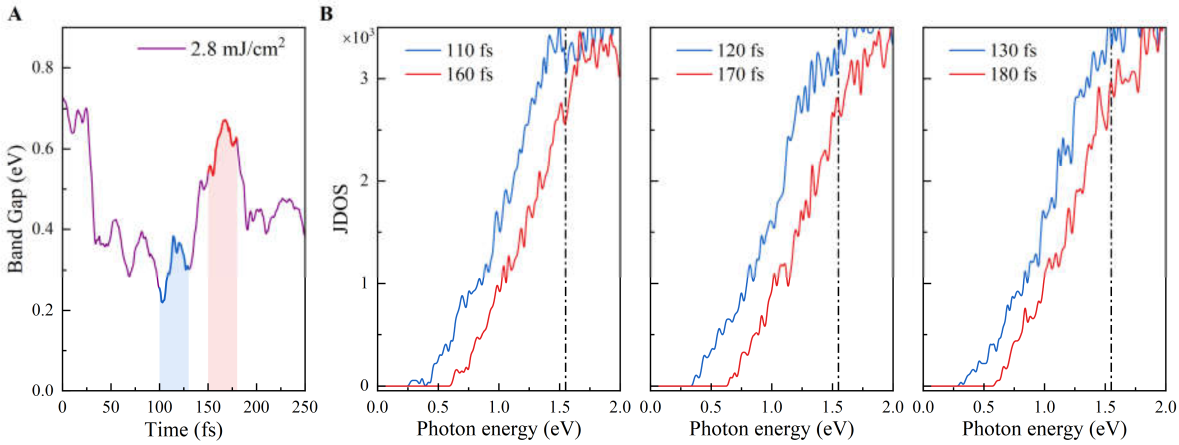

Fig. S9. Joint density of states (JDOS). (A) Temporal evolution of the bandgap under single-pulse excitation at 2.8 mJ/cm^2. The blue (delay time = 100 fs) and red (delay time = 150 fs) shaded regions indicate the time windows during which the Gaussian pulse acts on the system. **(B)** JDOS at 100-130 fs (blue line) and 150-180 fs (red line).

Section 4: Lattice temperature dependence of coherent and local disorder dynamics

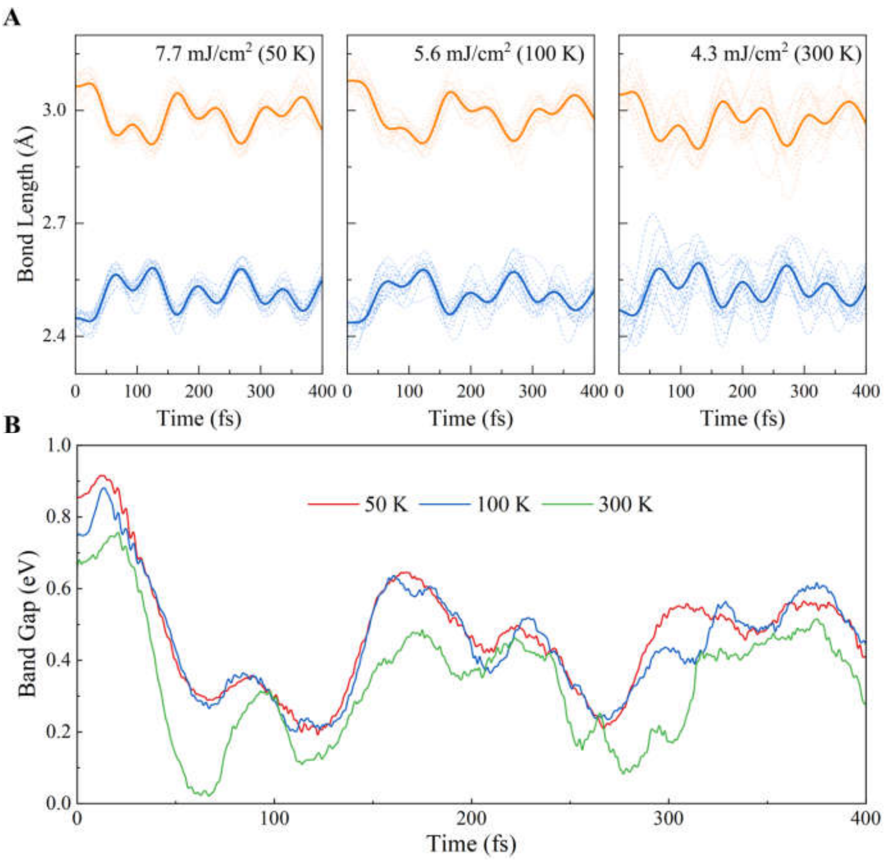

Fig. S10. Effect of temperature on coherent motion. (A) Time evolution of V-V bond lengths in the 96-atom system under photoexcitation, at initial temperatures of 50 K, 100 K, and 300 K. **(B)** The corresponding bandgap evolution at 50 K, 100 K, and 300 K.

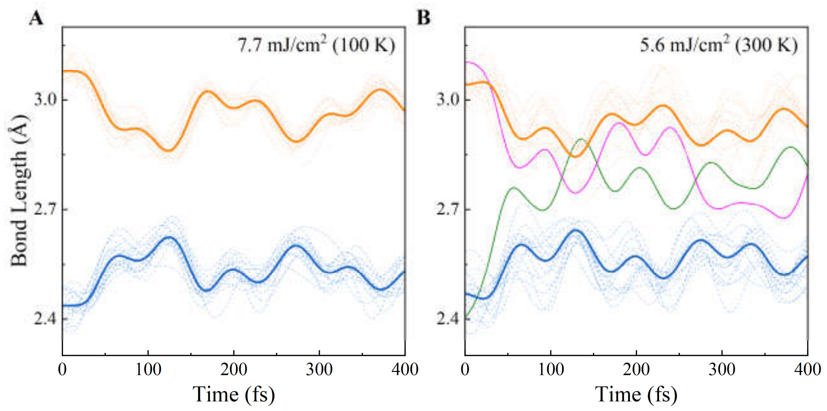

Fig. S11. Effect of temperature on the formation of localized effects. Time evolution of V-V bond lengths in the 96-atom system under photoexcitation, at initial temperatures of 100 K (**A**) and 300 K **(B)**. The pink and green lines indicate the formation of local polarons.

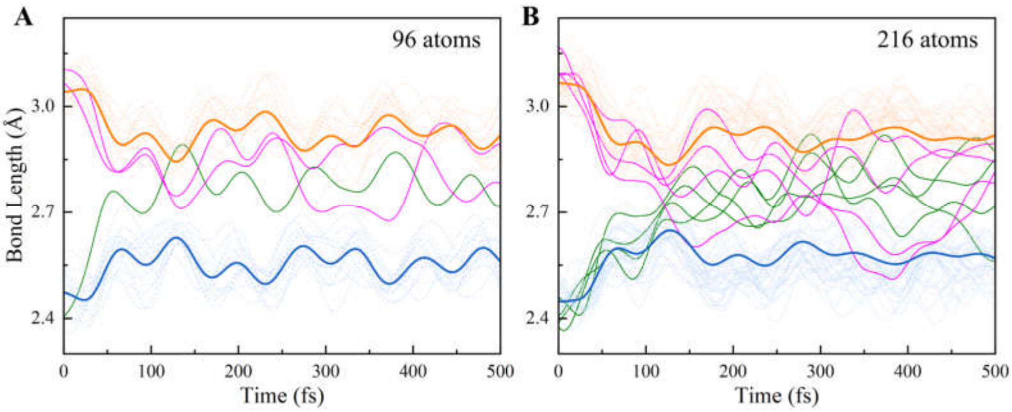

Fig. S12. Comparison of dynamics in 96-atom and 216-atom systems. Temporal evolution of the V-V bond lengths for systems containing **(A)** 96 atoms and **(B)** 216 atoms under the same photoexcitation fluence of 5.6 mJ/cm^2. The average lengths of the long and short V-V bonds are shown as orange and blue solid lines, respectively. The green and pink solid lines highlight the selected local bonds, while the orange and blue dashed lines indicate the other long and short bond lengths.

To assess the effect of supercell size on localized polarons, we performed simulations with 96-atom and 216-atom systems. Approximately 6% of the V-V dimers are broken in the 96-atom supercell, compared to about 6-8% in the 216-atom case (Fig. S12), indicating a proportional increase with system size. Therefore, in our study, the finite-size effect on photoexcited polarons is negligible. To improve computational efficiency and qualitatively assess the effect of lattice temperature, here we employ a reduced simulation setup using a 96-atom supercell with a 2×2×2 k-point sampling. The reduced system size does not affect the validity of our conclusions (Figs. S10 and S11).

Section 5: Effects of laser parameters on the dynamics

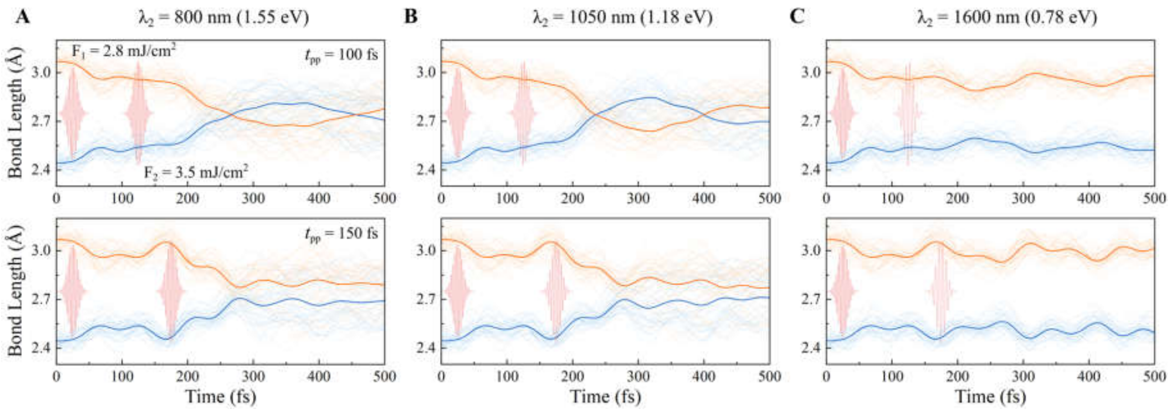

Fig. S13. Dependence of efficiency on laser wavelength in the case of coherent control. (A-C) Time evolution of V-V bond lengths under a first 50-fs pump pulse (800 nm, 2.8 mJ/cm^2), with an additional second 50-fs pump pulse (3.5 mJ/cm^2) applied at 100-fs and 150-fs delay. The wavelength of the second pulse ranges from 800 nm to 1600 nm.

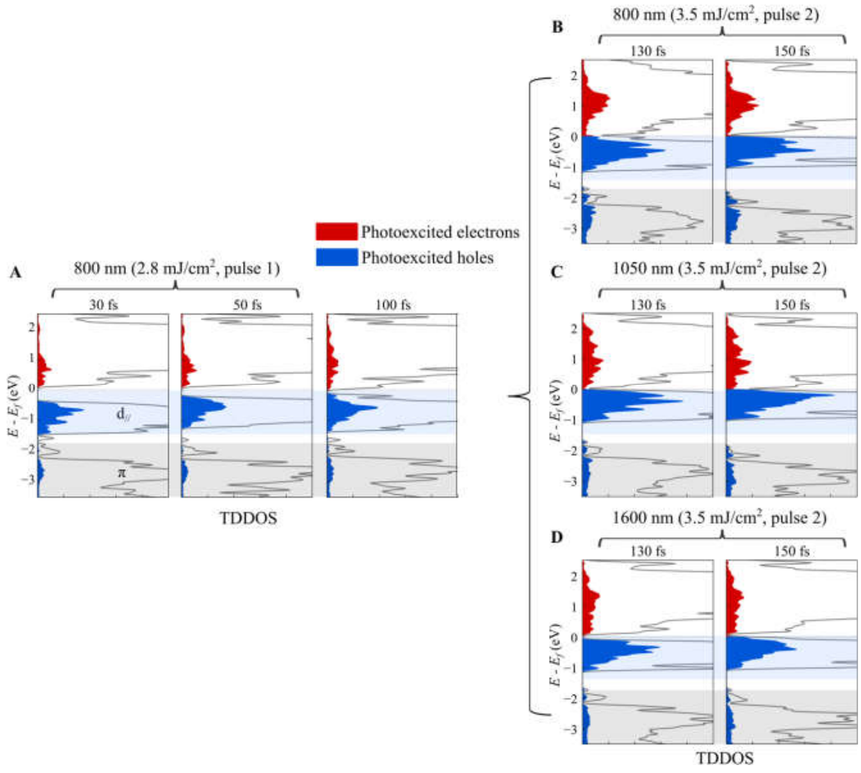

Fig. S14. Time-dependent density of states under double-pulse excitation with a 100 fs delay time. A first 50-fs pump pulse at 800 nm and 2.8 mJ/cm^2 is followed by a second 50-fs pulse at 3.5 mJ/cm^2, using 800 nm, 1050 nm and 1600 nm laser at 100 fs delay time. The red and blue shaded areas represent photoexcited electrons and holes, respectively. The light blue shaded region marks the energy range of $d_{||}$ orbital, while the light gray region corresponds to π orbital.

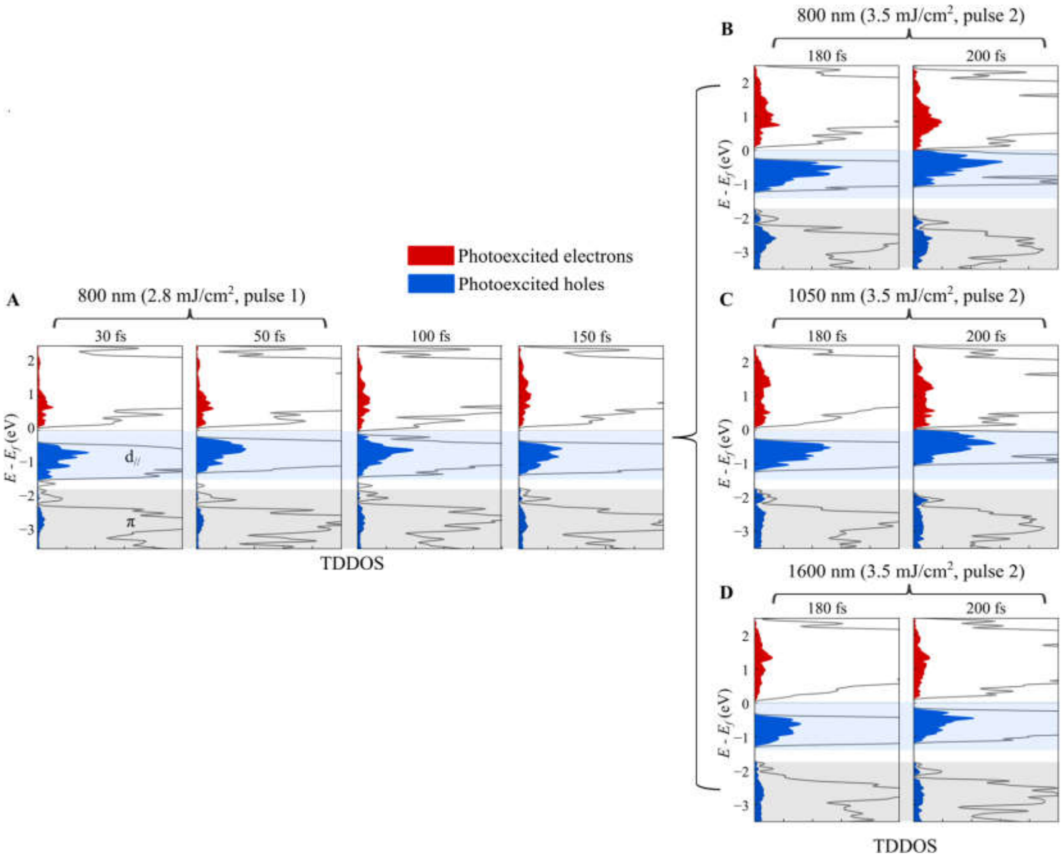

Fig. S15. Time-dependent density of states under double-pulse excitation with a 150 fs delay time. A first 50-fs pump pulse at 800 nm and 2.8 mJ/cm^2 is followed by a second 50-fs pulse at 3.5 mJ/cm^2, using 800 nm, 1050 nm and 1600 nm laser at 150 fs delay time. The red and blue shaded areas represent photoexcited electrons and holes, respectively. The light blue shaded region marks the energy range of $d_{||}$ orbital, while the light gray region corresponds to π orbital.

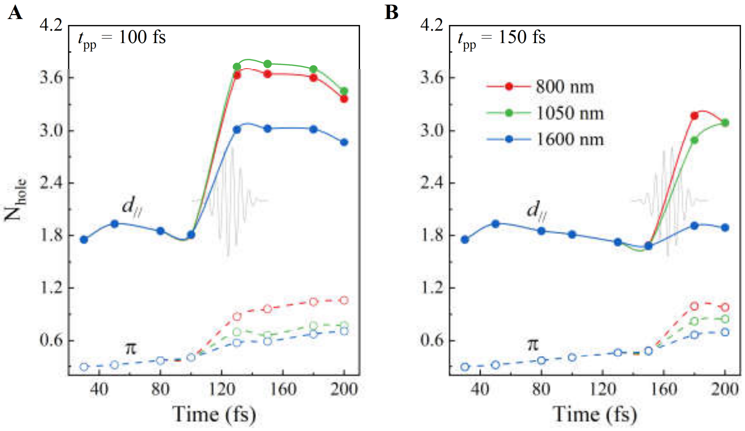

Fig. S16. Time evolution of photoexcited hole. (A, B) Time evolution of photoexcited hole populations in the $d_{||}$ orbital and π orbital under second-pump excitation (first pump pulse: 2.8 mJ/cm^2, second pulse: 3.5 mJ/cm^2) at 100 fs **(A)** and **(B)** 150 fs.

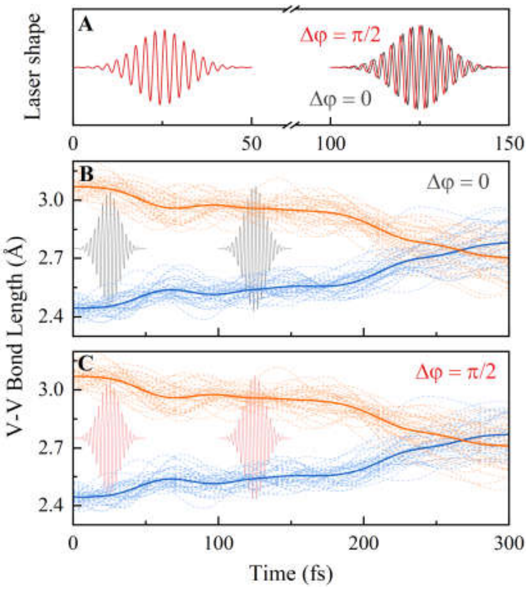

Fig. S17. Phase-clock effect of double-pulse excitation. Time evolution of V-V bond lengths under a first 50-fs pump pulse (2.8 mJ/cm^2, 800 nm), followed by a second 50-fs pulse (3.5 mJ/cm^2, 800 nm) applied at delay times of 100 fs. The phase difference between the two pulses is 0 (top) and π/2 (bottom).

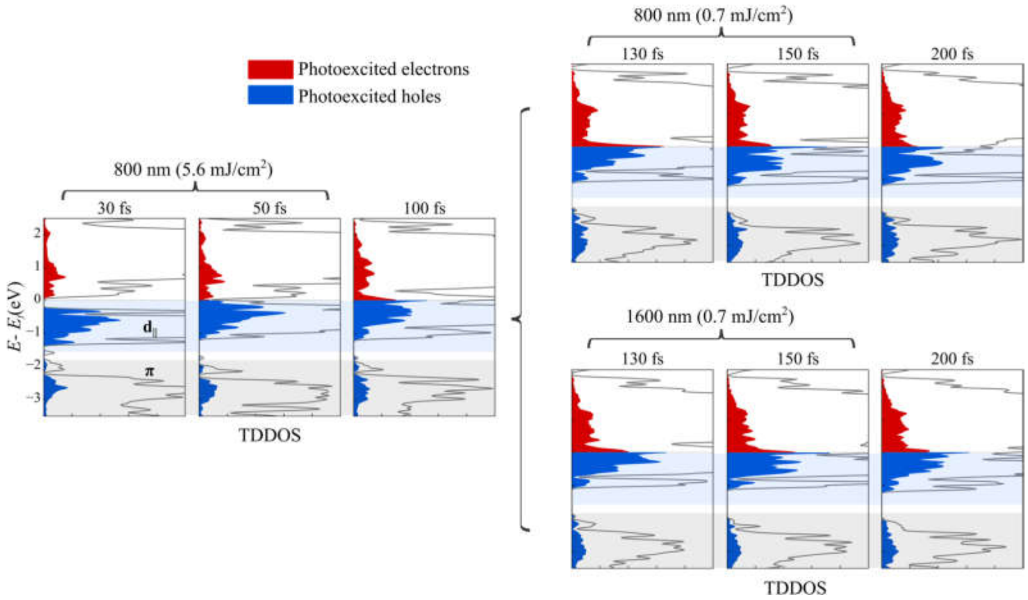

Fig. S18. Time-dependent density of states under double-pulse excitation. A first 50-fs pump pulse at 800 nm and 5.6 mJ/cm^2 is followed by a second 50-fs pulse at 0.7 mJ/cm^2, using either 800 nm (top panel) or 1600 nm (bottom panel). The red and blue shaded areas represent photoexcited electrons and holes, respectively. The light blue shaded region marks the energy range of $d_{||}$ orbital, while the light gray region corresponds to π orbital. After the second pulse, the population of $d_{||}$ holes is higher for 1600 nm excitation compared to 800 nm, and the π hole population is reduced. Lower photon energy (1600 nm) more effectively excites $d_{||}$ electrons.

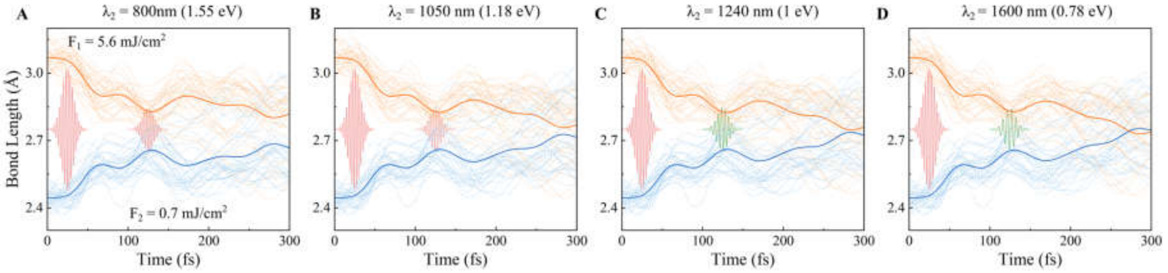

Fig. S19. Dependence of efficiency on laser wavelength in the case of localized phase transition. (A-D) Time evolution of V-V bond lengths under a first 50-fs pump pulse (800 nm, 5.6 mJ/cm^2), with an additional second 50-fs pump pulse (0.7 mJ/cm^2) applied at a 100-fs delay. The wavelength of the second pulse ranges from 800 nm to 1600 nm.

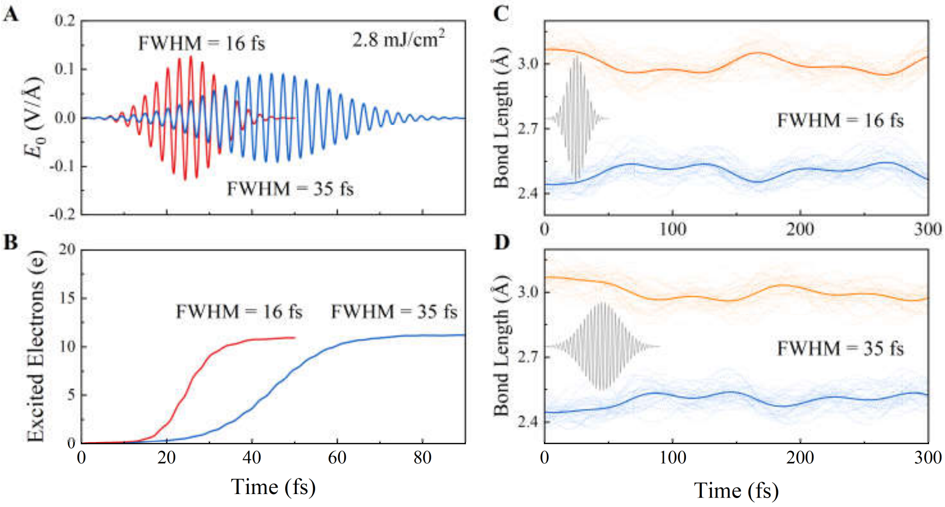

Fig. S20. Effect of pulse duration on phase transition. (A) Temporal profile of the external electric field applied to VO_2 at fluence of 2.8 mJ/cm^2. **(B)** Time evolution of the corresponding excited electrons. **(C-D)** Time evolution of the V-V bond lengths for pulses with full width at half maximum (FWHM) of 16 fs and 35 fs, respectively.
